# Whipple procedure: a review of a 7-year clinical experience in a referral center for hepatobiliary and pancreas diseases

**DOI:** 10.1186/s12957-015-0523-8

**Published:** 2015-03-11

**Authors:** Amir Saraee, Jalal Vahedian-Ardakani, Ehsan Saraee, Roshanak Pakzad, Massoud Baghai Wadji

**Affiliations:** Iran University of Medical Sciences, Firoozgar General Hospital Tehran, Tehran, Iran; Department of Surgery, Iran University of Medical Sciences, Firoozgar General Hospital, Tehran, Iran; Tehran University of Medical Sciences, Tehran, Iran; Shahed University of Medical Sciences, Tehran, Iran

**Keywords:** Pancreatic cancer, Whipple procedure, Periampullary carcinoma

## Abstract

**Background:**

Pancreatic cancer is generally found in the older population Pancreaticoduodenectomy seems to be the only way in resolving these resectable tumors. Allen. O Whipple was the first to describe pancreaticoduodenectomy in 1935 as a modified procedure. This article is a case series with respect to the 7-year experience of the Whipple procedure in Firoozgar Teaching Hospital.

**Methods:**

Patient surgery details were gathered from the surgical records of the operating room and their clinical records from the hospital archives. Data was analyzed with SPSS software (version 16.0.1). Those patients, whose tumor had invaded the superior mesenteric artery, had extensive portal vein involvement or distant metastasis was considered as unresectable.

**Results:**

The first Whipple procedure was recorded in our hospital in 2008. From 2008 till 20 March 2014, 70 cases were collected and analyzed. The mean age of cases was 58.4 years, the mean hospital stay length was 12.9 days (±6.23 days), mean operation time was 376 min (±37.3 min),. The most common presenting symptom was jaundice (78.6 %). Delayed gastric emptying was the most common post-operative complication. The most prevalent cause of reoperation was intra-abdominal abscess. Major morbidities of these patients consisted of cardiac arrhythmias (21.4%) and pneumonia (10%). Minor complications were wound infection (17.1%) and delayed gastric emptying (32.9%). The statistics revealed pancreatic anastomosis failure as 2.9% and a decrease in mortality rate from 50% during the first years of this study to 16% to 20% during the last years.

**Conclusions:**

In this case series, the time of operation decreased during the recent years .Analysis shows a correlation between operation time and pack cell transfused during the operation, but no correlation was found between operation time and post-operation hospitalization course. It is true that hospital setting, socioeconomic level of the patients including their compliance, and the expertise of the surgeons and surgical staff can have an influence on the result of this operation, but it seems that the magnitude of the surgical stress of this procedure and the (compromised) functional reserve of this patient population can be a notable factor influencing the outcome.

## Background

Pancreatic cancer is one of the most important causes of death in eastern countries and the fourth cause of death from cancer in the western hemisphere. Only a few percent of patients can survive from this condition for more than 5 years. Most patients present with an advance stage of the disease, and only in 10% to 20% of them the mass is resectable. There are some factors for predicting the prognosis, such as size of the tumor, degree of differentiation, status of resected lymph nodes, and the involvement of the resected margins. In some articles, the post-resection CA 19-9 level and tumor DNA content were also mentioned as prognostic factors. Among various treatments for pancreatic cancers, surgery was the only chance for cure [1].

Predisposing factors for pancreatic cancer remain unknown, but tobacco is the most probable reason. Risk increases with longer exposure to smoke and greater tobacco usage. There is some evidence that alcohol, coffee, and aspirin consumption are among the predisposing factors. Other risk factors are history of diabetes or chronic pancreatitis, chronic cirrhosis, and patients with blood type A, B, or AB. Only 5% to 10% of these patients have a positive familial history of pancreatic cancer [2].

For the first time, Allen. O Whipple described pancreaticoduodenectomy in the year 1935 when he modified the procedure that was performed before by Alessendro Codinivillan in Italy and Walter Keusch in Germany [3].

In a similar study at the Massachusetts hospital [4], pancreatic cancer cases that required surgery were followed and improvements in Whipple procedure were reported. The aim of this study is to evaluate the evolution of this procedure from 2008 to 2014 in our hospital and analyze the statistics pertaining to the presenting signs and symptoms, Whipple procedure, and the clinical post-operation courses.

## Methods

Data regarding the surgery was extracted from the archives of Firoozgar General Hospital, and further information was obtained from the medical records from 2008 to 2014. Data related to the ‘pancreaticoduodenectomy’ or ‘whipple’ procedures were gathered using a checklist designed for this study. The data was analyzed using the SPSS software (version 16.0.1). The parameters and variables of the study were patient demographic data, presenting symptoms, physical signs, past medical history, intra-operative parameters, post-operative complications, pathology, causes of post-operative death, cause of readmission, and cause of reoperation. Those patients, whose tumor had invaded the superior mesenteric artery, had extensive portal vein involvement, or distant metastasis was considered as unresectable. None of the patients received neoadjuvant chemotherapy.

## Results

A number of 70 procedures were performed till 2014 by three different surgeons. As a mean, our center performs 10 procedures per year but the number of procedures had increased from 2 operations in 2008 to 25 operations in 2014.

Analyses have shown the mean age of cases as 58.4 years with 40% of the gender being male. A portion of patients (35.1%) were greater than 65 years of age. The minimum age was 29; the oldest patient being 78 years old.

Prevalence of diabetes mellitus before surgery was 13.5%; among them, 5% were on medication with oral agents and 2.7% with insulin. Meanwhile, 91.9% of the diabetic patients were not receiving any medication. After the procedure, 27% of patients had new onset diabetes mellitus. In this case series, the percentage of alcohol consumers was 2.7%, and 21.6% of patients were smokers. One patient (2.7%) was addicted to opium.

About 13.5% of them had a positive family history of cancer, and of them, 32.5% had pancreatic cancer.

The most common presenting symptom was jaundice (78.6%). Among them, 70% complained of abdominal pain. Other symptoms in order of frequency are weight loss, anorexia, nausea, vomiting, and fever.

### Pathology

Eight and a half percent (8.5%) of cases had benign lesions, but the main indication for pancreaticoduodenectomy was malignancy in the pancreas. In this study, the most common pathology was adenocarcinoma; 62.2% of them were well differentiated.

The lymph nodes were involved by the tumor in 35.1% of cases. The surgical pathology studies were as follows: two patients had intestine type adenocarcinoma, four had neuroendocrine tumors, and in one case, granulocytosis paraganglioma was observed. A 29-year-old woman had duodenal large-cell malignant lymphoma (diffuse type). Her presenting symptoms were weight loss, anorexia, and vomiting.

There were benign lesions in 5.9% of cases, like adenomatous dysplasia of the duodenum. About 52.9% of malignancies were periampulary lesions, and 38.6% of them were neoplasms of the pancreas. Liver metastasis and abdominal wall seeding was considered as unresectable.

Among patients who underwent staging before surgery, stage IIB was the most common stage. Unfortunately, there was no sufficient data in the archives regarding the staging of the other 48 patients (Table 1).

**Table 1:** **Stage of tumor before operation**

**Stage of tumor before operation**	**Number**
IA	5
IB	2
IIA	3
IIB	10
III	2
No staging was present	48

### Operation course

The mean operation time was 376 min (±37.3 min), and intra-operative blood transfusion was approximately 1.84 pack cells each operation (Table 2).

**Table 2:** **Changes of operation time, used pack cell during operation, and post-hospitalization days during the time spent**

**Date**	**Mean (hours) ± SD operation time average**	**Used pack cell during operation**	**Post-op time hospitalization average mean (day) ± SD**
2008	4.9 ± 0.06	3	17 ± 0.7
2009	5.7 ± 1.12	6	17 ± 5.7
2010	5.0 ± 0.51	4	8 ± 6.4
2011	4.2 ± 0.53	2	10 ± 5.7
2012	5.0 ± 0.78	3	17 ± 13
2013	4.5 ± 0.33	2	15 ± 6.9
2014	5.2 ± 1.02	1	11 ± 5.2

The mean age of patients was 58.4 years; the mean admission length was 12.9 days (±6.23 days). Of them, 45.9% of patients suffered from abdominal pain, weight loss was seen in 16.2% of them, and 8.1% of them cases had foul stool after surgery.

The common technique for reconstruction in our study was classic pancreaticojejunostomy (83.8%). In one case, we repaired the transverse mesocolon that was injured inadvertently.

In some cases, the major vessels were involved by the tumor. If the SMA was involved, the case was considered inoperable and excluded from this study. SMV or portal vein could be repaired if less than 180° of the circumference of portal vein was involved by the tumor. In this case series, vascular reconstruction was performed in 13 patients (11.4% portal reconstruction, 7.1% SMV reconstruction). Vascular repair was performed due to vein involvement by tumor or iatrogenic injury.

### Complications

The most common complication after surgery was delayed gastric emptying (32.9%). GI leakage was related to failure of gastro-jejunostomy anastomosis, and biliary fistula was secondary to choledocojejunostomy anastomosis disruption or leakage. Major morbidities of these patients consisted of cardiac complications like cardiac arrhythmias (21.4%), pneumonia (10%), hemorrhage (7.1%), biliary fistula (2.9 %), and renal failure (5.7%). Minor complications were wound infection (17.1%) and delayed emptying (32.9 %) (Table 3).

**Table 3:** **Complications**

**Complications**	**Number**	**Percent**
Delayed emptying	23	32.9%
Cardiac complications	15	21.4%
Wound infection	12	17.1%
Pneumonia	7	10.0%
Hemorrhage	5	7.1%
GI leakage	5	7.1%
Renal failure	4	5.7%
Biliary fistula	2	2.9%
Pancreatic fistula	0	0.0%
Cholangitis	0	0.0%

### Reoperation

The most common cause for reoperation was intra-abdominal abscess (21.4%). Other causes of reoperation were bleeding in one case and obstruction in two. Biliary cutaneous fistula was seen in two patients, and one was operated twice due to bowel obstruction. Other causes of reoperation consist of wound dehiscence, tracheostomy, and gastrojejunostomy revision.

### Readmission

Thirty-seven (37%) percent of cases were readmitted due to various problems. After nausea and vomiting as the most common reason of readmission in 24.2% of cases, the second reason was pneumonia (19.24%). In order of decreasing frequency, other readmission causes were wound infection, controlling blood sugar, intra-abdominal abscess, GI bleeding, and fever.

### Post-operation course

In the early post-operative mortality group (death below 1 month after operation), seven cases died due to septic shock, three of them had massive gastrointestinal bleeding, and one patient had pulmonary thromboembolism (Table 4). The patients with septic shock were considered as a probable failure of pancreatic anastomosis since there was no site of infection in other organs, and autopsy was not allowed to be performed by one patient’s family.

**Table 4:** **Post-operative death**

**Early post-op death**	**Number**	**Percent**
Septic shock	7	10
Massive GI bleeding	3	4.3
PTE	1	1.4
Hypovolemic shock	1	1.4
ARDS	1	1.4
Acute MI	0	0.0

## Discussion

Pancreatic cancer is the fourth leading cause of cancer death because of short-term survival time, regardless of its stage. The main etiology of pancreatic cancer is unknown, though the incidence of pancreatic cancer is 2.5 to 3.6 times more in smokers. There also exists some evidence that alcohol, coffee, and aspirin consumption can lead to pancreatic cancer. The presenting symptoms of pancreatic cancer depend on the location, size, and stage of the tumor [2]. Pancreatic cancer is mostly seen in the elderly, and the overall 5-year survival rate is below 5% [5-7].

The histologic precursor best characterizing pancreatic cancer is intra-epithelial neoplasia [8]. Dens stroma termed as desmoplastic reaction in pathological assessment is also characteristic of pancreatic cancer [9].

Allen Oldfather Whipple modified the pancreaticoduodenectomy procedure in 1935 [3,10,11]. Surgical biliary and gastric bypass was superior to endoscopic intervention in terms of quality of life improvement and decreasing rate of re-hospitalization and re-intervention [12]. Nowadays, Whipple is an acceptable operation that is performed in various malignant and benign diseases of pancreas and periampulary area [13].

During the past 7 years of our experience in this procedure, an improvement in the different aspects of the technique including lesser time of operation, less intra-operative bleeding, less pancreatic anastomosis failure, and decrease in hospital staying days. Pancreatic anastomosis was performed in two layers with non-absorbable suture material and in an end-to-end and invagination technique. In some cases, omentum was used as a barrier between the site of pancreatic anastomosis and superior mesenteric vein.

Though the statistics reveal a pancreas anastomosis failure of 2.9% but due to the lack of autopsy findings, the authors consider it as 10% because of sepsis with unknown origin. There was decreasing in mortality rate from 50% during the first year of this study (among two patients) to 16% to 20% during the last years (among 25 patients).

The number of this procedure has increased in our center during the recent years due to developing an advanced gastrointestinal department and a referring system from that ward. Although complications of this surgery are still observed in our hospital, its decreased rate could be attributed to better surgical techniques, better post-operation intensive care, and improved surgical staff expertise and more accurate patient selection.

Post-operation hospitalization duration has decreased too except for in some complicated cases.

We found a meaningful correlation between operation time and packed blood cell transfused intra-operatively (*P* value <0.05) but no correlation has been found between operation time and the length of hospitalization.

According to the surgical pathologic study, four patients had well-differentiated neuroendocrine tumor (NET). The mean age of this group was 61.75 years, and the average hospitalization time was 13.5 days. Three of the four patients were female. It is notable that although the tumor had invaded the superior mesenteric vein or portal vein in two patients, none of them had jaundice. It could be assumed that NET could have enlarged bulk of mass without resulting in obstructive jaundice, but adenocarcinoma causes jaundice even with a smaller tumor size. The most common presenting sign of NET was vomiting and abdominal pain. In spite of their burden, these tumors could be resected easier than adenocarcinoma. Survival of this group was greater than other pathologies (average = 36 months). Two cases in this group survived for 72 and 60 months after surgery. None of them have expired till date.

Several studies have reported the effect of treating a high volume of patients on post-operative outcomes. The definition of high and low volume varied among all these studies from 15 patients to 40 patients per year.

Birkmeyer et al. have reported a marked difference in mortality rates of Whipple procedure in very low-volume (zero or one patient per year) and low-volume (one or two per year) hospitals compared with higher-volume hospitals (less than five per year). Surgeons who performed fewer than four resections per year had more complications than those who performed more than four [14].

On the other hand, mortality rates at very low and low-volume hospitals were significantly higher than those at high-volume hospitals.

## Conclusions

Though pancreaticoduodenectomy is the treatment of choice in the patients with periampulary carcinoma and in spite of its introduction to the medicine 80 years ago, it still has significant mortality and morbidity even in high volume centers.

It is true that hospital setting, socioeconomic level of the patients including their compliance, and the expertise of the surgeons and surgical staff can have influence over the result of this operation; it seems that the magnitude of the surgical stress of this technique and the compromised functional reserve of these patient population can be a notable factor influencing the outcome.

Even though our data strongly suggests - as other studies - that pancreatic resections should be carried out at institutions that perform a large number of them annually, it should be kept in mind whether the extent of operation can be reduced in the future.
